# Host and Environmental Specificity in Bacterial Communities Associated to Two Highly Invasive Marine Species (Genus *Asparagopsis*)

**DOI:** 10.3389/fmicb.2016.00559

**Published:** 2016-04-21

**Authors:** Tânia Aires, Ester A. Serrão, Aschwin H. Engelen

**Affiliations:** Centro de Ciências do Mar-CIMAR, Universidade do AlgarveFaro, Portugal

**Keywords:** *Asparagopsis* sp., bacterial communities, metagenomes, adaptation, coastal vs. off-shore areas, polluted vs. pristine environments, eco-physiological functioning bacteria

## Abstract

As habitats change due to global and local pressures, population resilience, and adaptive processes depend not only on their gene pools but also on their associated bacteria communities. The hologenome can play a determinant role in adaptive evolution of higher organisms that rely on their bacterial associates for vital processes. In this study, we focus on the associated bacteria of the two most invasive seaweeds in southwest Iberia (coastal mainland) and nearby offshore Atlantic islands, *Asparagopsis taxiformis* and *Asparagopsis armata*. Bacterial communities were characterized using 16S rRNA barcoding through 454 next generation sequencing and exploratory shotgun metagenomics to provide functional insights and a backbone for future functional studies. The bacterial community composition was clearly different between the two species *A. taxiformis* and *A. armata* and between continental and island habitats. The latter was mainly due to higher abundances of Acidimicrobiales, Sphingomonadales, Xanthomonadales, Myxococcales, and Alteromonadales on the continent. Metabolic assignments for these groups contained a higher number of reads in functions related to oxidative stress and resistance to toxic compounds, more precisely heavy metals. These results are in agreement with their usual association with hydrocarbon degradation and heavy-metals detoxification. In contrast, *A. taxiformis* from islands contained more bacteria related to oligotrophic environments which might putatively play a role in mineralization of dissolved organic matter. The higher number of functional assignments found in the metagenomes of *A. taxiformis* collected from Cape Verde Islands suggest a higher contribution of bacteria to compensate nutrient limitation in oligotrophic environments. Our results show that *Asparagopsis*-associated bacterial communities have host-specificity and are modulated by environmental conditions. Whether this environmental effect reflects the host's selective requirements or the locally available bacteria remains to be addressed. However, the known functional capacities of these bacterial communities indicate their potential for eco-physiological functions that could be valuable for the host fitness.

## Introduction

Stress-tolerance and adaptation in disturbed environments are mainly studied at the scale of populations of single species. Population genetic diversity is a key factor for adaptation to changing environments (e.g., Massa et al., 2013). The local gene pool influences a population's capacity to persist and to expand beyond the native range (for non-indigenous species—NIS) or as habitat changes (e.g., for edge populations; Pauls et al., 2013). Besides, most introduced/edge populations are only a subset of the entire species gene pool, often displaying very limited genetic diversity (Bridle and Vines, 2007; Dlugosch and Parker, 2008).

Driven by the rapid advance of culture-independent technologies, there is increasing evidence that the entire holobiome, involving distinct types of symbiosis between microorganisms and host eukaryotes, and the genetic richness of those microbial communities can play a determinant role both in adaptation and evolution of higher organisms (Zilber-Rosenberg and Rosenberg, 2008; Tonon et al., 2011; Dittami et al., 2014). In some cases, like the human gut microbiome, called the forgotten organ (O'Hara and Shanahan, 2006), the success of the host is so dependent on the associated microorganisms that the microbial genome functions as an extension of the genome of the host (Mandrioli and Manicardi, 2013). The hologenome theory of evolution considers that the holobiont and its hologenome (the sum of host and associated microbiota genome) act in consortium, as a unit of selection in evolution. Genetic variation in holobionts can arise from changes in either the host or the symbiotic microbiota genomes (Rosenberg et al., 2010). The diverse microbial symbiont community can aid the holobiont in surviving, multiplying and acquiring time necessary for the host genome to evolve and keep up with rapid and drastic environmental changes (Rosenberg et al., 2010). Some bacterial strains associated to invasive seaweeds and absent in the native range, are suggested to play a role in stress tolerance (Aires et al., 2013, 2015), as found in terrestrial plants.

Microbial communities establish stable associations with eukaryotic hosts, influencing host fitness and mutually fulfilling several crucial functions (Thompson et al., 2015). Bacterial assemblages associated with distinct marine eukaryotes include groups involved in important metabolic processes such as nitrification, nitrogen fixation (Chisholm et al., 1996), sulfate reduction (Crump and Koch, 2008), photosynthesis (Barott et al., 2011), plant growth enhancement (Orole and Adejumo, 2011), morphogenesis induction (Nakanishi and Nishijima, 1996), or chemical defense (Lee et al., 2009; Burke et al., 2011).

It is uncertain how bacterial assemblages organize across eukaryote hosts, what drives their organization and how stable those communities are. In the specific case of seaweeds, associated bacterial communities can be species-specific or even variety-specific (e.g., Aires et al., 2013, 2015), but they can also change seasonally (Lachnit et al., 2011), or result from a competitive lottery model where bacteria with similar metabolic abilities (functions) will be stochastically recruited (Burke et al., 2011). The interactive effects of both host and environment have only recently been discussed (Campbell et al., 2015; Marzinelli et al., 2015). Studies on corals and seaweeds suggested that bacterial assemblages determined by host specificity can be disrupted by environmental pressures (e.g., anthropogenic pollution; Marzinelli et al., 2015) and the host will tend to adapt to local conditions by selecting a more advantageous “hologenetic” background from the available bacterial guild (Kelly et al., 2014). However, species-specificity disruption can make the host more prone to diseases (Morrow et al., 2012).

Among the species included in the lists of the “worst invasive alien species threatening biodiversity in Europe” (EEA, 2007) and the Mediterranean Sea are those in the red seaweed genus *Asparagopsis* Montagne (Bonnemaisoniales, Rhodophyta) (Streftaris and Zenetos, 2006; Andreakis et al., 2007) that has been spreading rapidly across European waters (Andreakis et al., 2009). Although, with contrasting geographical distributions, together, *A. armata* and *A. taxiformis* are present along all continents and in all oceans across the world, partially due to multiple introduction events (Andreakis et al., 2004; Sherwood, 2008). *A. taxiformis* is considered cosmopolitan in subtropical and tropical communities worldwide (Abbott, 1999) and, so far, five cryptic lineages have been described for this species, with distinct geographic distributions (Dijoux et al., 2014). Due to its complexity and cryptic nature, its natural vs. invasive distribution is still under discussion for some parts of the globe (Dijoux et al., 2014). The more temperate species *A. armata* consists of two cryptic lineages naturally distributed along western and southern Australia and New Zealand and non-indigenous in the Northeast Atlantic and Mediterranean coasts (Andreakis et al., 2007; Dijoux et al., 2014).

In this study, we characterize bacterial communities associated to both *Asparagopsis* species in contrasting environments: mainland (coastal) and islands (offshore, where only *A. taxiformis* is present) across the southern Northeast Atlantic, where both species are described as invasive.

With a cosmopolitan distribution and, consequent, high adaptive potential (despite the presumably low genetic variability inherent to invasive species), these species represent a good model to study holobiont adaptation to different environments and anthropogenic influences. Considering the whole genetic pool (host + associated bacteria) and expecting a more prompt response from the bacterial partners when compared to that of the host (Rosenberg et al., 2010) we believe that the integration of this genetic component will provide innovative insights and the right tools to anticipate the spread of invasive species.

Bacterial communities are characterized through next generation barcoding of the V5–V8 region of 16S rRNA gene, in combination with exploratory functional assessments using shotgun metagenomics. Based on previous studies in other marine organisms (e.g., Burke et al., 2011; Aires et al., 2015), we make the following predictions: (1) host-associated bacterial communities will cluster differentially according to host species if the host species plays an important role in the association; (2) part of the host-associated bacterial guild will be habitat dependent, if they are necessary for coping with the habitat requirements; (3) if the host has “habitat-related” functional requirements fulfilled by the microbiome, then these should be mirrored in the functional profiles of the associated bacteria; (4) if associated bacterial communities (after subtraction of the environmentally available community) are assembled solely according to a lottery model, then none of the above hypotheses should be confirmed. In this dynamic perspective of the holobiont, both host and environment would be decisive for the dynamics of the holobiont structure and the “habitat-dependent” part might offer, to the host, some resilience to disturbed habitats.

## Materials and methods

### Sampling and DNA extraction

**Figure 1:**
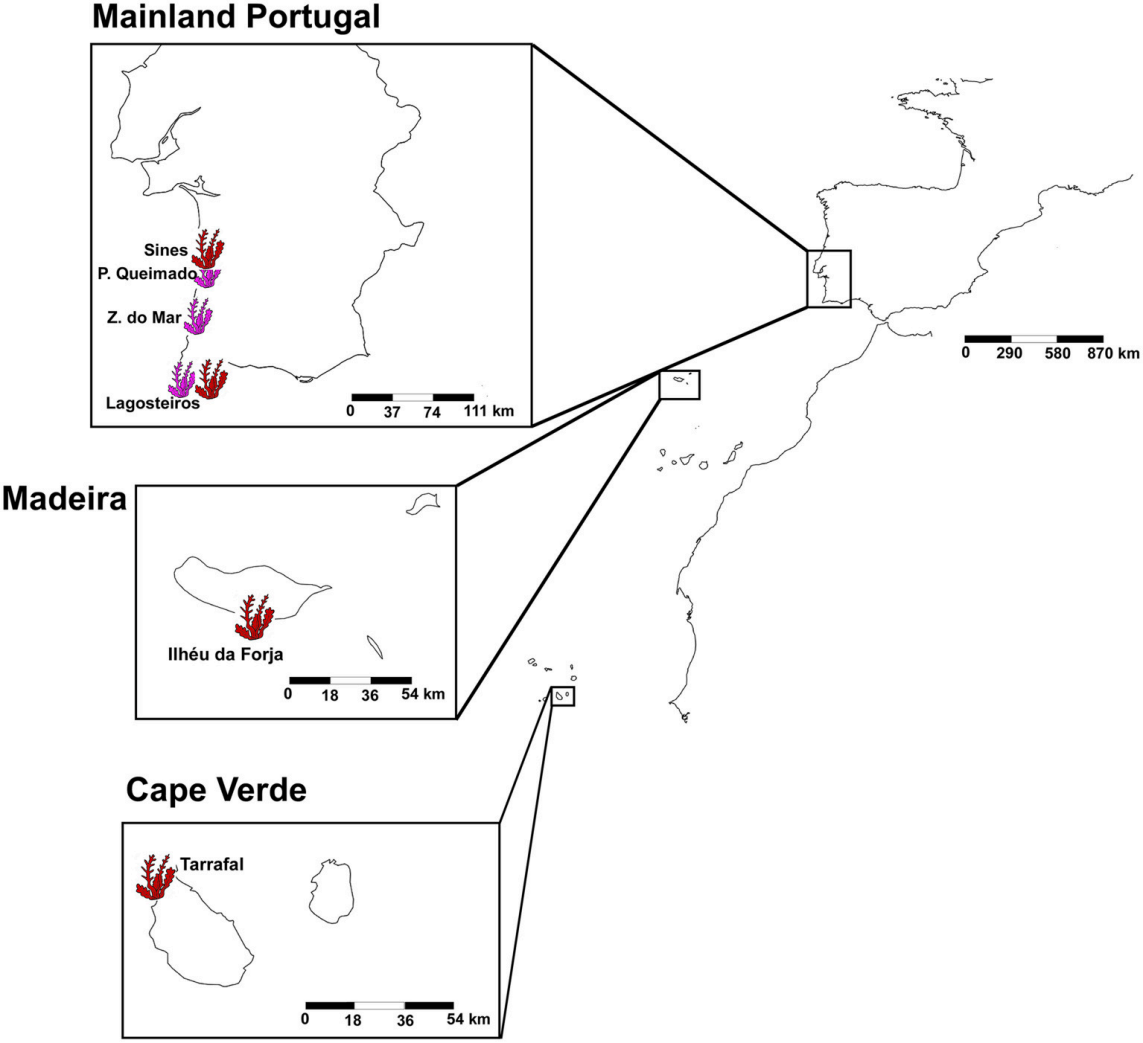
**Sampling locations of *Asparagopsis taxiformis* (in pink) and *Asparagopsis armata* (in red)**. *Asparagopsis taxiformis* was sampled in Ilhéu da Forja (Madeira Island), Sines and Lagosteiros (Mainland Portugal) and in Tarrafal Beach (Cape Verde). *Asparagopsis armata* was sampled in three mainland locations: Lagosteiros, Praia do Queimado, and Zambujeira do Mar.

A total of 32 samples of the two *Asparagopsis* species, *A. armata*, and *A. taxiformis*, were analyzed from six different locations in the south-east of the northern Atlantic ocean (Figure 1). The more temperate species, *A. armata*, was sampled at three sites on the southwest coast of mainland Portugal (Praia do Queimado (N37°49.22448 W-8°47.5671), Zambujeira do Mar (N37°27.81342 W-8°47.8779), and Lagosteiros (N37°0.6201 W-8°55.79388), Figure 1). *A. taxiformis* was sampled in one location of two different Islands: Santiago Island in Cape Verde (Tarrafal Beach—N15°16.85736 W-23°45.1824), and Madeira Island in Portugal (Ilhéu da Forja—N32°38.14746 W-16°55.8099); and on the mainland coast of Portugal at the only locations where it was known to exist: Sines (N37°56.9361 W-8°52.79724) and Lagosteiros (N37°0.6201 W-8°55.79388; Figure 1). Due to its absence in the offshore islands, *A. armata* sampling was restricted to mainland areas. In this part of the Atlantic both species seldom occur in sympatry, Lagosteiros is the only exception we are aware of. For each location, *A. taxiformis* was represented by four replicates and *A. armata* by six replicates (with the exception of Lagosteiros where only four replicates were successful in the end). Replicated sediment and seawater samples (*n* = 8) were taken as environmental control. Seawater was filtered in the field using 0.2 μm filters, which were stored the same way as the other samples. Tissue and environmental samples were flash frozen in liquid nitrogen, in the field, immediately after sampling and kept at −80°C until DNA extraction. Samples were unfrozen and cleaned of attached particles and immediately put in the PowerSoil® DNA Isolation Kit (MO BIO, Laboratories, Inc.) extraction lysis solution. Bacterial DNA extraction, for all samples was done following the MO BIO Vortex Adapter protocol.

### Bacterial tag-encoded 16S amplicon sequencing

The total 16S rRNA region was amplified using the universal primers 27F and 1492r (Lane, 1991) with the following changes to the original protocol: after an initial denaturation at 95°C for 2 min, conditions were as follows: 35 cycles of denaturation at 95°C for 20 s, annealing at 55°C for 20 s, and extension at 72°C for 90 s. The final extension was at 72°C for 3 min. The 25 μl reaction mixture contained 250 μM dNTPs, 0.6 μM of each primer, 1 × 2 PCR buffer mix, 2 μl of template DNA (with a final concentration of about 10 ng μl^−1^) and 0.3 μl of Taq polymerase (Advantage® 2 Clontech). PCR products were cleaned using ExoFastAP enzyme following the manufacturer protocol (Thermo Scientific™) and amplified DNA was processed by Biocant (Cantanhede, Portugal) using a nested-PCR prior to sequencing. Modified 8 bp key-tagged primer 799F-mod3 along with the reverse primer 1392R (Region V5-V8 of the 16S), which avoid chloroplast cross amplification (Hanshew et al., 2013), were used and PCR conditions were as follows: 95°C for 3 min, 10 cycles of 95°C for 20 s, 50°C for 30 s, 72°C for 30 s, and a final elongation of 72°C for 3 min. Tagged amplicons from modified partial 16S rRNA amplification were analyzed through Pyrosequencing, GS FLX Titanium, 454-Life Sciences-Roche technology®.

### Bacterial community characterization based on 16S rRNA diversity

All samples were sequenced in the same run and generated tagged pyrosequences were trimmed for quality with the standard sff software tools from Roche454. In the outsourced company, sequences were screened for a minimum read length of 500 bp and <2 undetermined nucleotides. All the diversity analyses were performed using the program QIIME 1.6.0: Quantitative Insights Into Microbial Ecology (Caporaso et al., 2010). All the samples, barcoded with unique tags, were analyzed together as a single dataset. The filtered dataset containing only high quality sequences was filtered through a conservative chimera detection using the ChimeraSlayer method (Haas et al., 2011). Selected high quality sequences were clustered into Operational Taxonomic Units (OTUs) within reads using the UCLUST module from QIIME and a pairwise identity threshold of 0.97. Representative sequences for each OTU were picked using the “most-abundant” method and OTU sequence alignment was performed with Pynast (Caporaso et al., 2010). The Ribosomal Database Project (RDP) classifier was used for taxonomic assignment with a 97% confidence threshold. To assign each OTU to the closest matching described taxon, searches were performed against the SILVA database (Quast et al., 2013) and sequences were putatively assigned to a described taxon if the *e*-value exceeded a minimum threshold of 0.001 (default value). Sequences with the best match for eukaryotes (i.e., chloroplasts and mitochondria) were excluded from the OTU table in downstream analyses as well as unassigned sequences.

#### Alpha-diversity measures

After removal of chimeras, eukaryotic sequences, unassigned sequences, and rare OTUs (global singletons removal, defined as OTUs that only occurred once in the entire data set), OTUs common among seaweeds and environmental samples (sediment and seawater) were removed in order to obtain OTUs unique for the seaweeds (this was the dataset used for all the analysis). Interesting comparisons between environmental exclusive OTUs across sampling locations were not possible due to unsuccessful amplification of environmental samples from the Islands.

After OTU table normalization to the minimum number of sequences (after common to environment OTUs removal), Chao I richness (Chao, 1984), Shannon index and number of distinct OTUs were calculated to assess diversity values within samples (α-Diversity) using alpha_diversity.py script on Qiime (Caporaso et al., 2010). Analyses of variance (One-way ANOVA) were performed to access significant differences in alpha-diversities among *Asparagopsis* groups (*A. armata* mainland, *A. taxiformis* Islands, and *A. taxiformis* mainland) using QI Macros SPC Software for Excel. Turkey's HSD post hoc test was performed when differences were significant, in order to identify between which groups differences occurred. Additionally, Rank abundance curves for each *Asparagopsis* group were calculated in Qiime (Caporaso et al., 2010), using logarithmic relative abundances.

#### Bacterial community structure and characterization (β-diversity)

Using the normalized (rarefied) data set, diversity between the different samples (β-Diversity) was estimated using Bray-Curtis dissimilarity measure to build the distance matrix (Bray and Curtis, 1957) after square root transformation of the data. PERMANOVA was used to test for differences between samples with the *a priori* factors: Type of Sample (seaweed vs. environmental), species, and location. One-Way Analyses of similarity (ANOSIM) were performed in order to test for differences within “groups” (*A. armata* Mainland, *A. taxiformis* Islands and *A. taxiformis* Mainland). For ANOSIM, significance levels (*p*-values) and factors strength (*R*-values) values were calculated to assess the similarity within and between groups. The samples were considered statistically different when, both, the *p*-values were inferior to 0.05 and the *R*-values were close to one. Canonical analysis of principal coordinates (CAP), based on Bray-Curtis matrix (from rarefied OTUs table), was used to test assignment/clustering of *Asparagopsis* samples with *a priori* factors (groups- species/Mainland vs. Islands). Discriminant vectors with a Pearson correlation >0.7 were considered important. The number of vectors was reduced to represent each bacterial genus/family/order/class by a single vector [i.e., OTUs assigned to the same taxonomic level were left with just one representative (the one with the highest absolute abundance)—for each group]. All analyses were performed using PRIMER-E+PERMANOVA v.6 (Clarke and Warwick, 1994). To identify OTUs specific and shared among defined sample groups a Venn-Diagram was constructed using Venny 2.0 (Oliveros, 2007). The diagram was constructed with the total number of OTUs (after removing shared OTUs between seaweeds and environment) and following the procedure as in Ainsworth et al. (2015) using only OTUs present in at least 30% of the replicates of a group. OTUs with a relative abundance higher than 1.5% were plotted by pooling them by order (for those unique to each group or shared among all groups). Metadata was submitted to MG-RAST (for accession numbers see Table 1).

**Table 1:** **Number of OTUs and sequences of each replicate after quality control and removal of chimeras, chloroplast, unassigned sequences, and singletons**.

**Species**	**Location**	**Site**	**Before comm. environment removal**		**After comm. environment removal**		**% OTUS shared**	**Normalization**	**MG-RAST accession numbers**
			**Number of OTUs**	**Number of sequences**	**Number of OTUs**	**Number of sequences**		**Number of OTUs**	
*A. armata*	SW Portugal (M)	Praia do Queimado	647	4989	407	1894	37.09	316	4673245.3
*A. armata*	SW Portugal (M)	Praia do Queimado	866	3993	610	2122	29.56	462	4673233.3
*A. armata*	SW Portugal (M)	Praia do Queimado	564	4685	355	1784	37.06	274	4673211.3
*A. armata*	SW Portugal (M)	Praia do Queimado	1266	5636	743	2291	41.31	532	4673229.3
*A. armata*	SW Portugal (M)	Praia do Queimado	744	5199	523	2348	29.70	344	4673251.3
*A. armata*	SW Portugal (M)	Praia do Queimado	870	4067	533	2140	38.74	404	4673231.3
*A. armata*	SW Portugal (M)	Zambujeira do Mar	695	3262	462	1576	33.53	408	4673264.3
*A. armata*	SW Portugal (M)	Zambujeira do Mar	1028	4168	630	2025	38.72	486	4673242.3
*A. armata*	SW Portugal (M)	Zambujeira do Mar	808	3709	521	1930	35.52	480	4673221.3
*A. armata*	SW Portugal (M)	Zambujeira do Mar	1171	4517	665	2112	43.21	479	4673255.3
*A. armata*	SW Portugal (M)	Zambujeira do Mar	1043	4745	668	2293	35.95	478	4673203.3
*A. armata*	SW Portugal (M)	Zambujeira do Mar	1142	4672	697	2380	38.97	402	4673207.3
*A. armata*	S Portugal (M)	Lagosteiros	1682	14687	1283	7359	23.72	459	4673253.3
*A. armata*	S Portugal (M)	Lagosteiros	1691	17809	1211	7850	28.39	413	4673219.3
*A. armata*	S Portugal (M)	Lagosteiros	1655	13961	1046	6604	36.80	436	4673261.3
*A. armata*	S Portugal (M)	Lagosteiros	1619	11938	1202	6753	25.76	485	4673259.3
*A. taxiformis*	Cape Verde (Santiago Island)	Tarrafal	752	4182	511	1987	32.05	410	4673217.3
*A. taxiformis*	Cape Verde (Santiago Island)	Tarrafal	902	4379	607	2415	32.71	450	4673254.3
*A. taxiformis*	Cape Verde (Santiago Island)	Tarrafal	425	3867	288	1784	32.24	256	4673215.3
*A. taxiformis*	Cape Verde (Santiago Island)	Tarrafal	239	3393	176	2163	26.36	140	4673213.3
*A. taxiformis*	Portugal (Madeira)	Ilhéu da Forja	889	5296	586	3397	34.08	345	4673262.3
*A. taxiformis*	Portugal (Madeira)	Ilhéu da Forja	991	6588	579	3981	41.57	334	4673239.3
*A. taxiformis*	Portugal (Madeira)	Ilhéu da Forja	1144	5591	729	3251	36.28	459	4673206.3
*A. taxiformis*	Portugal (Madeira)	Ilhéu da Forja	1124	5933	608	2936	45.91	393	4673243.3
*A. taxiformis*	S Portugal (M)	Lagosteiros	1664	12571	933	4420	43.93	492	4673260.3
*A. taxiformis*	S Portugal (M)	Lagosteiros	2386	18002	1312	6487	45.01	546	4673227.3
*A. taxiformis*	S Portugal (M)	Lagosteiros	1323	5539	633	1680	52.15	561	4673225.3
*A. taxiformis*	S Portugal (M)	Lagosteiros	999	3632	429	1383	57.06	417	4673256.3
*A. taxiformis*	SW Portugal (M)	Sines	1918	9644	1009	4709	47.39	468	4673258.3
*A. taxiformis*	SW Portugal (M)	Sines	2041	15481	1136	9126	44.34	333	4673241.3
*A. taxiformis*	SW Portugal (M)	Sines	963	4499	451	2405	53.17	307	4673244.3
*A. taxiformis*	SW Portugal (M)	Sines	1253	4707	561	1883	55.23	461	4673235.3
Sediment	SW Portugal (M)	Praia do Queimado	1677	6099	564	1944	–	227	4673247.3
Sediment	SW Portugal (M)	Zambujeira do Mar	1503	5469	566	2006	–	240	4673237.3
Sediment	S Portugal (M)	Lagosteiros	1136	4236	360	1170	–	236	4673223.3
Sediment	SW Portugal (M)	Sines	262	4111	88	1345	–	88	4673257.3
Seawater	SW Portugal (M)	Praia do Queimado	941	3975	325	1002	–	204	4673263.3
Seawater	SW Portugal (M)	Zambujeira do Mar	773	4129	298	1218	–	167	4673209.3
Seawater	S Portugal (M)	Lagosteiros	271	3431	125	1155	–	80	4673250.3
Seawater	SW Portugal (M)	Sines	1317	3470	359	1055	–	238	4673252.3

*Before and after removal of OTUs common to environmental (sediment and seawater) samples is shown as well as the % of OTUs shared between each replicate and environmental samples. Number of OTUs after normalization to the minimum number of sequences (after common to environment removed—1364) is also presented*.

*M, Mainland; SW, Southwest, S, South*.

### Functional profiles based on metagenomics

In order to provide the first functional insights of the associated bacterial communities, three metagenomes were sequenced each consisting of five pooled samples of: (1) *A. armata* from Praia do Queimado (mainland); (2) *A. taxiformis* from Sines marina (mainland), and (3) *A. taxiformis* from Tarrafal beach, Cape Verde (islands), after DNA extraction (following the same procedure as above). The decision of pooling samples from the same location was due DNA concentration of individual samples below those required for metagenomics.

Metagenome sequencing of the samples was carried out at Molecular Research (MR DNA), Shallowater, Texas, using MiSeq 2 × 150 bp (Illumina) sequencing. 50 ng of DNA from each sample was used to prepare the libraries using Nextera DNA Sample Preparation Kit (Illumina). Following the amplification and denaturation steps, libraries were pooled and sequenced. Library insert size was determined by Experion Automated Electrophoresis Station (Bio-Rad). Pooled library (12 pM) was loaded to a 600 Cycles v3 Reagent cartridge (Illumina) and sequencing was performed on Miseq (illumina). As joining paired-end reads was only successful for a very low percentage of reads (2.5–9.5%), sequences >60 bp from only one paired-end were used. Samples were analyzed utilizing MG-RAST metagenome analysis server (Meyer et al., 2008). Metagenomic sequence reads were compared with the SEED protein database (http://theseed.org/wiki/Main_Page) using BLASTx. For functional annotation, sequences were assigned the function of the closest identified protein and these functions were grouped into metabolic pathways according to the subsystems in the SEED database, at an *E*-value cutoff: 1 × 10^E−5^ minimum identity of 60%, and a minimum alignment length of 15 measured in aa for protein and bp for RNA databases. Predicted genes were tabulated and classified into functional categories from lower levels (individual genes) to higher levels (cellular processes). Using MG-RAST automatic tools, a heatmap was constructed to depict the normalized data of the most abundant level 1 subsystem categories in the SEED database and access the similarity/differentiation among the three different groups. Data has been normalized in MG-RAST. The normalization procedure includes two steps, applied independently, to each metagenomic sample: transformation and standardization. A third step, multiple sample scaling, is applied to all considered data (i.e., is applied simultaneously to all samples under consideration). In this last step, after each sample has undergone transformation and standardization, the values for all considered samples are scaled from 0 (the minimum value of all considered samples) to 1 (the maximum value of all considered samples). This is a uniform scaling that does not affect the relative differences of values within a single sample or between/among two or more samples (Meyer et al., 2008). These three MG-RAST Normalization steps reduce bias caused by differences in sequencing depths.

Samples were clustered in a dendrogram using Ward's minimum variance method with Bray-Curtis distance metric in normalized values. Candidate metabolisms that could provide insight into the different functional profiles of both environments (Mainland and off-shore islands) were chosen, and deeper levels (level 2 and 3) were analyzed for differential metabolisms, so from the total metabolic assignments and not just those represented in the heatmap. The metagenomes are publicly available in MG-RAST database (4638179.3—*A. taxiformis* Mainland, 4638178.3—*A. taxiformis* Cape Verde island, 4636013.3—*A. armata* Mainland).

## Results

### Bacterial community diversity and characterization based on 16S rRNA diversity

The complete dataset resulted in a total of 276174 high-quality sequences (after quality control, removal of chimeras, chloroplast, and unassigned sequences), along with a total of 28838 unique OTUs (Accession Number—KU615570—KU639569). For the analysis, we have removed singletons, which left us with a total of 260261 sequences and 12925 unique OTUs (Table 1 and Table S1). After the removal of OTUs shared between seaweeds and environmental samples (ranging roughly from 23–57%—Table 1) the number of sequences declined to 118363 with a correspondent number of unique OTUs of 9877 (Table 1 and Table S1). Due to the high variation in the number of sequence among replicates (1002–8649 sequences), the dataset was normalized to the minimum number of sequences (1002) and used, as such, for further analysis.

**Figure 2:**
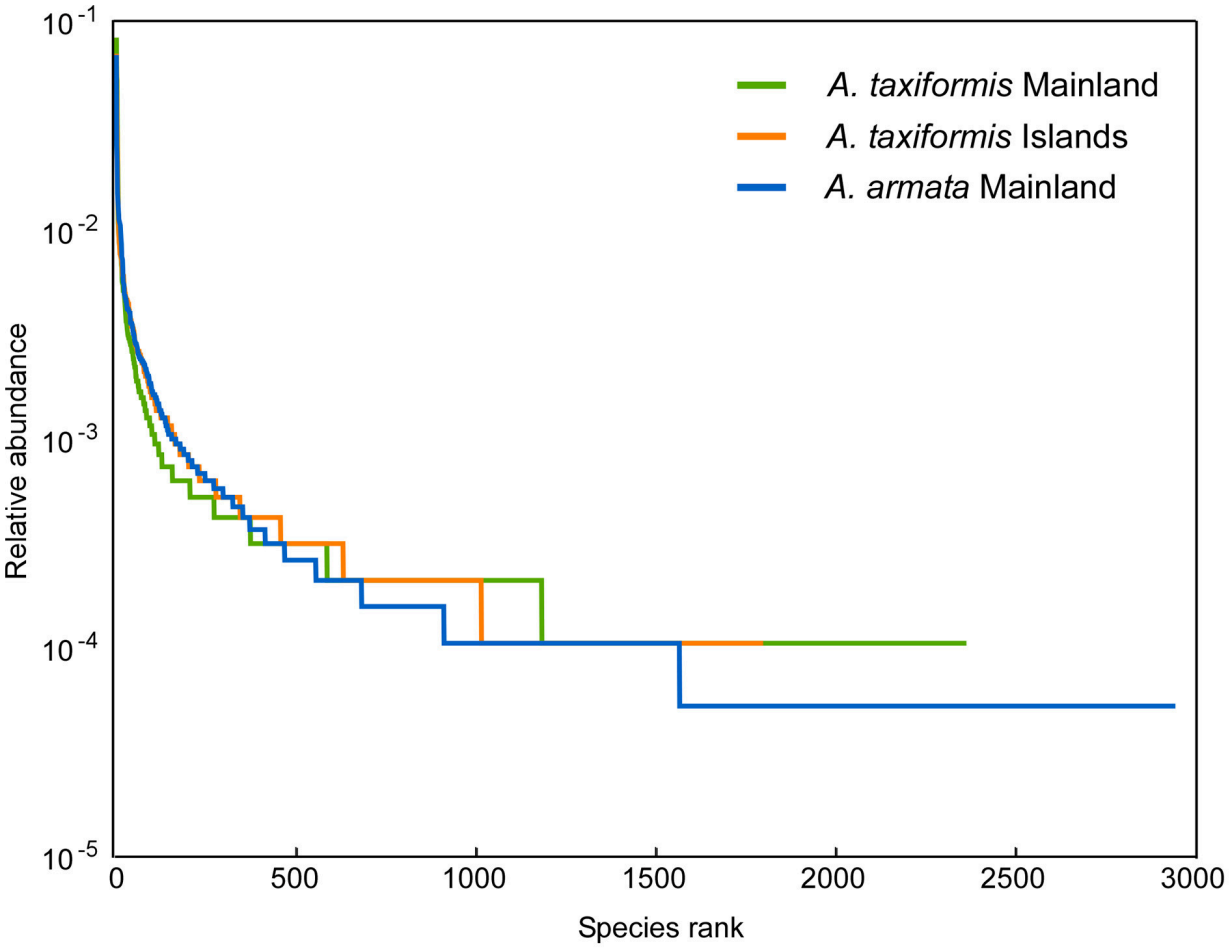
**Rank abundance curves for each *Asparagopsis* group plotting logarithmic relative abundances**.

Reduced α-diversity values (Table S3) in *Asparagopsis* from islands compared to mainland groups was detected for the richness index, Chao1 (*p* = 0.0059, Table S4A) and not for the Shannon index or the number of distinct OTUs (*p* = 0.7–0.06, Tables S4B,C). Once the removal of singletons may bias Chao1 calculation, these results were compared with those from the dataset with singletons (not shown) and the main conclusions were the same, not affecting the ANOVA results. The slope of Logarithmic rank abundance curves was steep for all three groups representing a low evenness and *A. taxiformis* from the Islands showed the lowest species rank/ richness (Figure 2).

PERMANOVA main test showed significant differences for the factors “species” and “location” (*p* = 0.001, Table 2). Significant differences were found between the two species at the only location they were sampled together (Lagosteiros, *p* = 0.026, Table 3A). Within the factor species, *A. armata*, showed no differences among the different locations (*p* = 0.139–0.370, Table 3B), whereas *A. taxiformis* presented significant differences between insular and mainland locations (*p* = 0.005–0.009, Table 3B).

**Table 2:** **Results of PERMANOVA main test with Square root transformation and Bray-Curtis distances**.

**Source of Variation**	**Pseudo-F**	***P* (*perm*)**
Species	3.5182	**0.001**
Location	3.3461	**0.001**
Sp × Lo	No test	No test

***H_0_**: there are no differences in the distribution of OTUs in different species and locations, H_0_ rejected if: **p < 0.05**. Factors abbreviations: Sp, Species; Lo, Location, Bold face values are significant at P < 0.05, P-values based on 999 permutations*.

**Table 3:** **(A) Pairwise PERMANOVA comparisons for species within Lagosteiros using Monte Carlo 999 simulations (MC). (B) Pairwise PERMANOVA comparisons for Locations within each *Asparagopsis* species using Monte Carlo 999 simulations (MC)**.

**A**				
**Within Level “Lagosteiros” of Factor “Location”**				
P (MC) (*t*-statistic)		*A.taxiformis*			
*A.armata*		**0.026** (1.9385)			
**B**				
**Within Level “*****A. armata*****” of Factor “Species”**				
*t*-statistic P (MC)	Praia do Queimado	Zambujeira do Mar	Lagosteiros	
Praia do Queimado		0.139	0.370	
Zambujeira do Mar	1.255		0.231	
Lagosteiros	1.030	1.151		
**Within Level “*****A. taxiformis*****” of Factor “Species”**				
*t*-statistic P (MC)	Lagosteiros	Sines	Cape Verde	Madeira
Lagosteiros		0.104	**0.008**	**0.005**
Sines	1.717		**0.009**	**0.006**
Cape Verde	2.183	2.212		0.060
Madeira	2.339	2.462	1.736	

*Significant p-values (alpha = 0.05) shown in bold. **H_0_**: there are no differences in the distribution of OTUs in the different levels of the different factors, H_0_ rejected if: **p < 0.05**. Bold face values are significant at P < 0.05*.

As bacterial communities of the two different islands did not show significant differences in composition (*p* = 0.060; Table 3B), they were pooled as “*A. taxiformis* Islands” hereafter. ANOSIM analysis comparing the hereafter designated “groups” (*A. armata* Mainland, *A. taxiformis* Islands and *A. taxiformis* Mainland), showed significant differences among all groups (*p* = 0.0010–0.0020, Table 4) with strong dissimilarity among groups (*R* = 0.708–0.984). All groups were significantly different from environmental samples (*p* = 0.0010–0.0018, Table 4).

**Table 4:** **Statistical results of One-way ANOSIM with Bray-Curtis distance measures applied to each *Asparagopsis* group of species using OTU hits (Based on 9999 permutations)**.

	***A. taxiformis mainland***	***A. armata***	**Sediment**	**Seawater**	***A. taxiformis* islands**
*A. taxiformis*		0.001	0.0018	0.0018	0.002
Mainland					
*A. armata*	0.984		0.001	0.001	0.001
Sediment	0.882	0.998		0.6503^a^^*^	0.002
Seawater	0.984	1	0.792^a^		0.002
*A. taxiformis*	0.926	0.708	0.987	0.943	
Islands					

*P-values are represented on the top of the matrix and R values on the bottom*.

***H_0_**: there are no differences in the distribution of OTUs between the members of the various groups, H_0_ rejected if: **p < 0.05**. R-values vary between 0 and 1. Large positive R (up to 1) signifies dissimilarity between groups*.

* *There are no significant differences between the groups*.

a *Significance (p) value is >0.05 but R-value shows high separation between levels of factors*.

**Figure 3:**
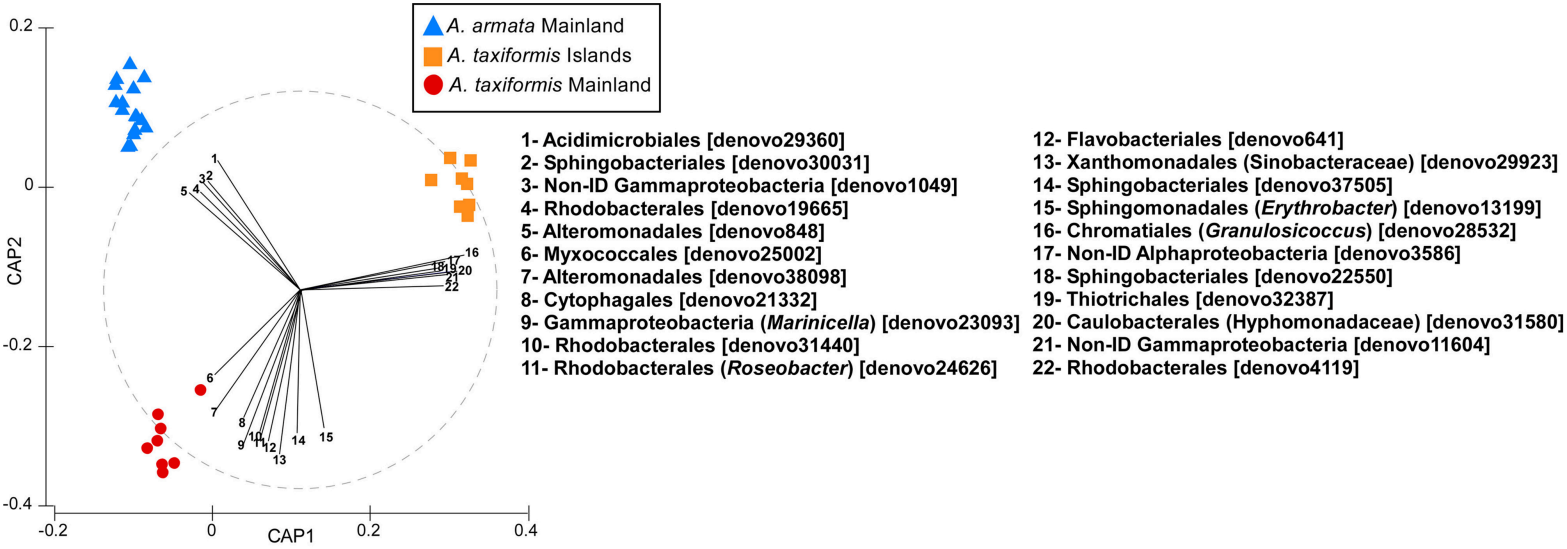
**Canonical analysis of Principle coordinates (CAP) plot showing the bacterial communities compositions of the main *Asparagopsis* groups (delimited according to previous statistical analyses)**. OTUs with Pearson's correlation >0.70 have been overlaid on the plot as vectors. Graphic was changed as described in the main text.

As clearly illustrated in the Canonical Analysis of Principal Coordinates (CAP), *Asparagopsis* associated bacterial communities reflected their host group/taxa specificity (*A. armata* and *A. taxiformis p* = 0.0010; Table 2, Figure 3, these results are strengthened by the significant differences between the species when occurring in sympatry—Lagosteiros—*p* = 0.026, Table 3A), however also a strong oceanic effect is reflected in the grouping “*A. taxiformis* Islands” vs. “*A. taxiformis* Mainland” (pairwise *p* = 0.0020; Table 4, Figure 3). The vectorial correlations visualized within the CAP (Figure 3) clearly showed some OTUs that are more correlated to the above mentioned groups. The *A. armata* bacterial community was characterized by a low number of discriminative/highly correlated OTUs and only Acidimicrobiales were exclusive (in the CAP analysis) to this group (Figure 3). Mainland *A. taxiformis* showed a higher correlation (when compared with the other groups) with Myxococcales, Alteromonadales, Cytophagales, Gammaproteobacteria from the *Marinicella* genus, Sphingomonadales from the *Erythrobacter* genus, Xanthomonadales from the Sinobacteraceae family, Rhodobacterales from the *Roseobacter* genus, and Flavobacteriales (Figure 3). In contrast, *A. taxiformis* from the oceanic islands had a higher relative abundance of OTUs assigned to Thiotrichales and Caulobacterales from the Hyphomonadaceae family, Chromatiales from the *Granulosicoccus* genus. Abundance of represented OTUs for each replicate, within each group, is shown in Table S2.

Intersection among groups of samples, through a Venn diagram, showed that more OTUs were shared between habitat than between species: *A. taxiformis* Mainland and *A. armata* contained 892 shared OTUs, whereas *A. taxiformis* from Mainland and Islands shared only 251 OTUs (Figure S1). The number of unique OTUs for each group was: *A. armata*—2493, *A. taxiformis* Mainland—2196 and *A. taxiformis* Islands—1032 (Figure S1). After selecting the OTUs present in at least 30% of the replicates the proportion of OTUs shared between different species and between different habitats remained the same (147 vs. 38; Figure S1). The number of OTUs for the *A. armata* group decreased more than those of the other groups, which indicated a higher number of low abundance or unevenly distributed OTUs associated to *A. armata* (Figure S1). Of all the OTUs shared among all groups, 163 OTUs had <1% and only three had >5% relative abundance. From the 291 unique *A. armata* OTUs 288 had <1% relative abundance and only three had >5% relative abundance. The group of 357 OTUs unique for *A. taxiformis* Islands showed 354 OTUs with <1% relative abundance and three with >5% relative abundance. For those 822 unique for the *A. taxiformis* mainland group, 813 OTUs had <1% relative abundance, six OTUs had relative abundances between 1 and 3% and one OTUs showed a relative abundance of 34.5%. The taxa found to be more correlated to a certain group (mentioned above, Figure 3), were compared to the individual plots composed by unique OTUs for each group to make sure that they were represented and abundant for these groups and only those were used for discussion. Xanthomonadales had relative abundance <1.5%, but appeared four times as a highly correlated vector exclusively for the *A. taxiformis* mainland group. Shared OTUs were unevenly represented across the different groups (Figure S2).

### Functional profiles based on metagenomes

Shotgun sequencing resulted in a total of ~460 million quality reads with an average length of 100 bp, with more specifically 1,314,364 sequences and 1929 functional assignments for mainland *A. taxiformis*, 1,838,276 sequences and 369 functional assignments for the same species in Cape Verde Island and 1,453,236 sequences and 1870 functional assignments for mainland *A. armata*.

Only a low percentage of sequences was assigned to eukaryotes (0.7–1.4%).

**Figure 4:**
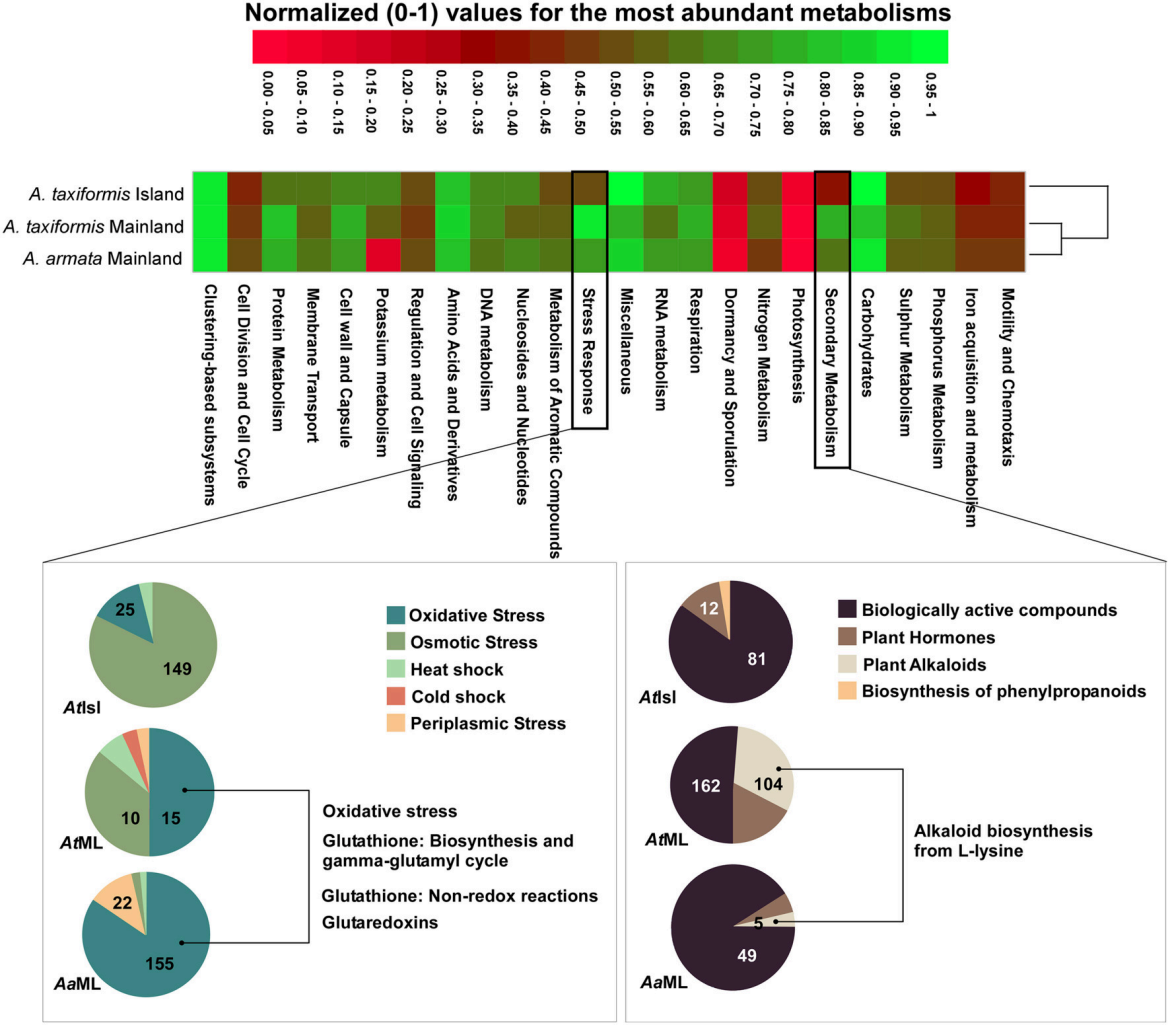
**Differential clustering of the most abundant metabolisms of *A. armata* (mainland) and *A. taxiformis* (mainland and island)**. Normalized (following MG-RAST normalization procedure) values were used to draw the heatmap. The hierarchical dendrogram was clustered using Ward's minimum variance method with Bray-Curtis distance metric. Level 2 metabolisms of the most abundant level 1 metabolic assignments for Mainland samples (compared to island samples) were represented in pie charts and level 3, of the most relevant metabolism from level 2, was described. Absolute numbers of hits for the most representative functions are represented in the corresponding slice. *Aa*ML- *A. armata* mainland, *At*ML- *A. taxiformis* mainland, *At*Isl- *A. taxiformis* Cape Verde. Graphics were manipulated to fit in the same figure (heatmap+pie charts) and pie charts colors were changed so the same feature color would match for all the samples (Raw data in Table S5).

Due to the lack of replicates for metagenomic data, we restrict ourselves to exploratory descriptions. The most abundant metabolic subsystem similarities showed that metabolisms are more similar between environments (island vs. mainland) rather than species (*A. armata* vs. *A. taxiformis*; Figure 4, raw data in Table S5).

Within the metabolic assignments, relative abundances for the “Mainland group” showed the highest values for protein metabolism, cell wall, and capsule, sulfur metabolism, secondary metabolism and stress response (Figure 4). The last two were investigated deeper due to their possible relation with more unstable/stressful environments (as in habitats under anthropogenic influence). Secondary metabolism showed biologically active compounds as the most abundant for all the samples, but alkaloids were only detected in mainland *A. taxiformis* (with 100% hits in alkaloid biosynthesis from L-lysine; Figure 4). Concerning stress response, “oxidative stress” is the predominant assignment for the “mainland group” with related metabolic assignments distributed through: oxidative stress, Glutathione: biosynthesis and gamma-glutamyl cycle, and Glutathione: non-redox reactions and glutaredoxins. In contrast, *A. taxiformis* from Cape Verde Island had more proportional assignments of metabolic subsystems to osmotic stress (82.32%; Figure 4).

**Figure 5:**
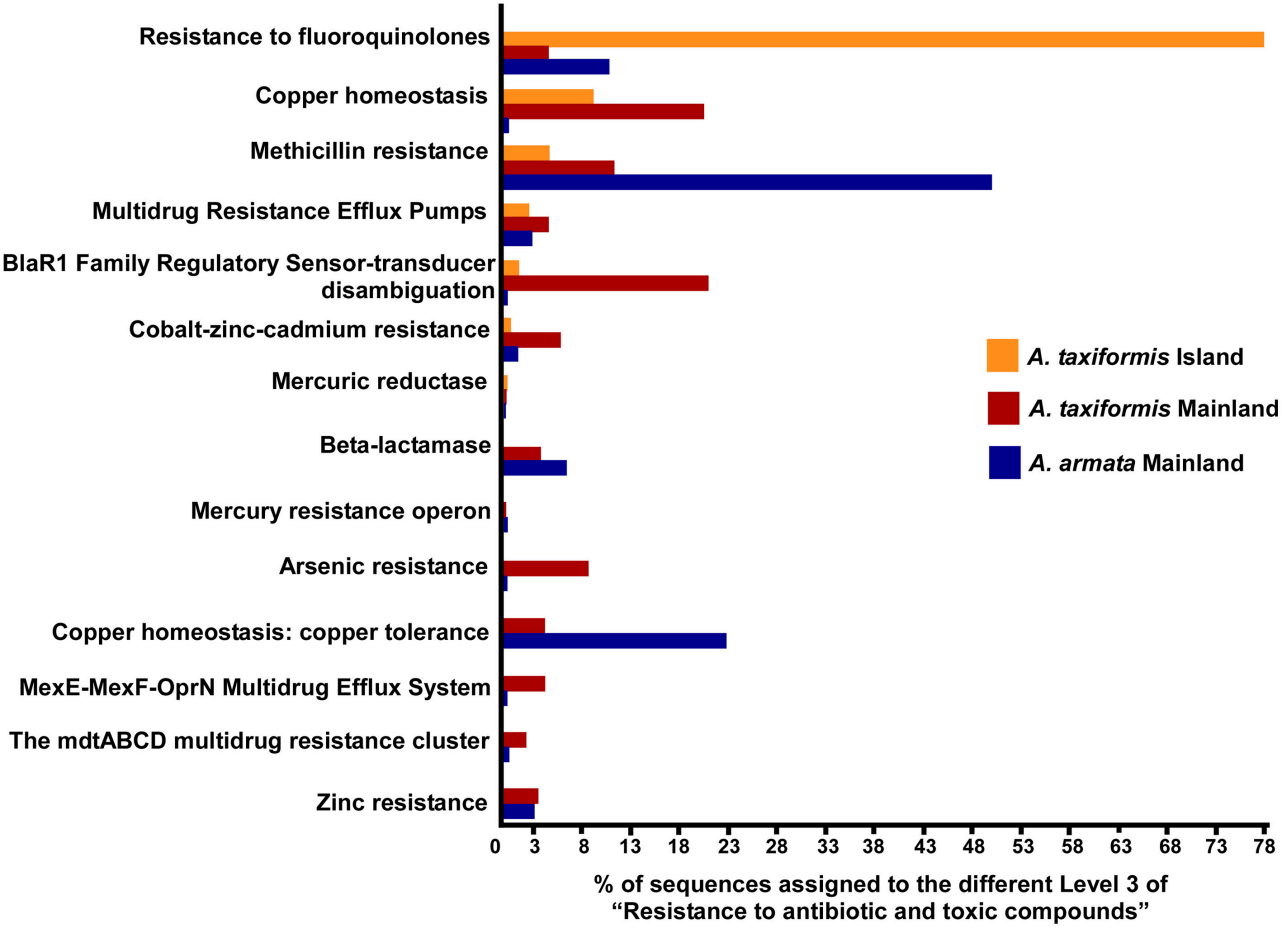
**Percentage of metagenomic sequences of functional composition of Level 2—Resistance to antibiotic and toxic compounds (assigned to the general SEED subsystems)—of *Asparagopsis* sps. from Mainland and Island (Raw data in Table S6)**.

Although not represented within the most abundant metabolic subsystems in the heatmap, resistance to antibiotic, and toxic compounds (from virulence, disease, and defense) was also investigated as a good candidate to distinguishing stress-related environments (Figure 5, raw data in Table S6). Around 50% of the metabolic assignments in resistance to antibiotic and toxic compounds were found in *A. taxiformis* from Cape Verde with a higher proportion on the Resistance to fluoroquinolones (78.7%). For *Asparagopsis* mainland, a much larger range of metabolic assignments (within resistance to antibiotic and toxic compounds) was found compared to the island samples. Mainland *A. armata* had most hits on Methicillin resistance (50.2%) and copper tolerance (23.2%) and mainland *A. taxiformis* showed more homogeneity among all the metabolic assignments with most hits for copper homeostasis (20.7%) and BlaR1 Family Regulatory Sensor-transducer Disambiguation (21.1%). Together, these results stress the wide range of assignments related to tolerance/resistance to heavy metals in mainland *Asparagopsis* (from Sines region) with relatively high number of hits for *A. taxiformis* (namely: Arsenic resistance—8.8%, Cobalt-zinc-cadmium resistance—6.0%, Zinc resistance—3.4% and copper tolerance—4.4%).

## Discussion

This study is the first report of species and environmental specificity of bacterial communities of a cosmopolitan/invasive species in contrasting habitats (Mainland vs. offshore Islands), using metabarcoding and metagenomics. Our results showed that the genus *Asparagopsis* carries bacterial communities that are well differentiated between the closely related sister species *A. taxiformis* and *A. armata* within the same habitat. However, the species-specific community composition of *A. taxiformis* showed a striking differentiation associated with contrasting environments (Mainland vs. offshore Islands). These results are novel and unexpected because other studies of invasive seaweed species have shown that hosts tend to maintain their specific bacterial community in invaded vs. native ranges thousands of Km apart, showing a strong consistent species-specific pattern (Aires et al., 2015). Yet, geographical versus environmental specificity are not contradictory or exclusive; as in contrast with Aires et al. (2013), here we compared contrasting different environments (coastal vs. offshore, polluted/ anthropogenically disturbed vs. pristine). Likewise, in the green alga *Bryopsis*, differences in bacterial communities were largely due to environment (13%) and host phylogeny (10%), while geography only explained 2% (Hollants et al., 2013).

Our functional profiles are in agreement with Burke et al. (2011) who found that there is functional genetic profile equivalence even when bacterial community composition is different. We show that the two different species (sharing the same environmental pressures) are closer in the dendrogram than the conspecific groups. In addition, in our study, bacterial community taxonomy also shows some overlapping in addition to the functional profiles. However, for a direct comparison with these authors' study, a more detailed and replicated approach would be needed.

The shared OTUs that could be considered closest to a core bacterial community were highly unevenly represented across the three groups and mostly mirrored the individual plots for groups' unique OTUs. The most widely shared bacteria, with high abundances for all groups, were members of the order Sphingobacteriales (see Table S1 and Figure S2) and might be a structural part of the *Asparagopsis* microbiome. In contrast, several other bacteria strongly contributed to the observed differentiation (Figure 3, Figure S2) showing high correlation to the different groups. Even though functional assignments based on 16S rDNA barcoding have to be done cautiously, some putative functions can be related to bacteria taxonomic classification, which we based on similar studies.

Bacterial community composition (associated with different organisms) has been shown to change when facing stress. The orders Acidimicrobiales, Sphingomonadales, Xanthomonadales, and Myxococcales increased in the rhizosphere of halophyte plants (*Halimione portulacoides* and *Sarcocornia perennis* ssp. *perennis*), in estuarine salt marshes in Portugal, when the concentration of hydrocarbons in sediment increased along with the presence of homologous genes related to OH degradation (Oliveira et al., 2014). Acidimicrobiales and Sphingomonadales were, in our case, part of mainland groups, but absent from the islands groups (Figure 3), whereas Xanthomonadales and Myxococcales were more dominant in the *A. taxiformis* mainland group. Specifically, 100% of the Sphingomonadales order were assigned to the *Erythrobacter* genus commonly detected in petroleum-contaminated soil, groundwater, and coastal seawater (Alonso-Gutiérrez et al., 2009). Besides, the genus *Roseobacter* (only highly correlated with *A. taxiformis* Mainland samples) was found to be closely related to oil-metabolizing functions in polluted environments (McKew et al., 2007). The Alteromonodales order was only found as distinguishing factor in the two coastal groups (Figure 3). This is in accordance with previous studies where bacterial communities associated with the polychaete *Ophelina* sp. react to the increase of heavy metals by an increase of abundance of bacteria from the Alteromonadales order (Neave et al., 2012). Likewise, Alteromonodales have also been found related to sites affected by urbanization and eutrophication (Marcial Gomes et al., 2008; Zeng et al., 2010) and some of it members are metal-resistant and capable of binding Cu^2+^ and Zn^2+^ cations thereby reducing their toxicity (Vincent et al., 1994).

The described functions of the groups here found are in agreement with our metagenomic data which showed a higher proportion of metabolic functions associated to stress response and resistance to toxic compounds, more specifically, most hits on resistance or tolerance to heavy metals (as copper, cobalt, arsenic, and zinc) in *Asparagopsis* from the mainland sites. Also, in seaweeds, the toxic effect of heavy metals, and other environmental stresses, appears to be related to production of reactive oxygen species (ROS), which impose oxidative stress on the cells (Dring, 2006) and results in unbalanced cellular redox status. Algae respond to heavy metals by induction of several antioxidants, including diverse enzymes such as glutaredoxins and the synthesis of low molecular weight compounds such as glutathione (Pinto et al., 2003; Mellado et al., 2012). This was reflected in our metagenomic data which showed functional assignments to the glutathione pathway, only found in mainland samples (where heavy metals and other environmental stresses are expected to be higher, which leads to ROS) which is used to scavenge the ROS produced (Rijstenbil et al., 2000; Ratkevicius et al., 2003).

Only samples collected in mainland showed hits on alkaloids. As an extra mechanism of defense, alkaloids are produced to enable plant/seaweed protection against pathogens and herbivores (War et al., 2012) and as detoxifiers in polluted soils by terrestrial plants (Khodjaniyazov, 2012). Genes of secondary metabolites, other than alkaloids, were also more abundant in our samples presumably more-exposed to pollution. It has been suggested that macroalgae depleted from their own chemical defense are able to rely on the secondary metabolites produced by their associated bacteria (Egan et al., 2000). Since the number of metagenome reads assigned to eukaryotes was very low, it is likely that these functions inferred were provided by the bacterial community.

All these results suggest complex interactions between macro and microorganisms. It is likely that that part of the seaweed microbiome might be more under influence of the environment rather than the host. However, there is also a degree of specific association as shown when invasive seaweed species switch environments yet maintaining their own specific microbiome (Aires et al., 2015).

Regardless of the lack of specific data on environmental variables of our sampling sites, the mainland coastal area, where our samples were taken, is under constant anthropogenic influence with fishing/commercial and recreational maritime activities. Also, due to the proximity of an industrial area (including a hydroelectric plant, refineries, and petrochemical industries) and a fishing port, the levels of pollutants are known to be high (Anonymous, 2008). The discriminative presence of the referred orders in the coastal groups, together with the higher metabolic assignments on protection mechanisms, might well be related with the host necessity of “gathering specific symbionts” with “specific functional capacities” for environmental remediation in order to survive and persist.

Nevertheless, some of these bacteria may be just looking up for themselves and their necessity of coping with the adversities. The hypothesis that the seaweed would not take advantage of the described mechanisms, cannot be discarded and should also be considered.

Bacterial communities associated to *A. taxiformis* from offshore Islands were distinguished by the presence of the family Hyphomonadaceae of the Caulobacterales order (in the combination of the results shown in Figure 3 and Figure S2). Hyphomonadaceae members are widely distributed in marine environments (Anast and Smit, 1988) and especially common in oligotrophic waters (Alain et al., 2008). They are believed to play an important role in the mineralization of dissolved organic matter (Abraham et al., 1999), an important trait in oligotrophic conditions (Biddanda et al., 2001). These studies are in agreement with our findings for the *A. taxiformis* Islands group sampled from oligotrophic/non-eutrophic waters.

The Island group show a distinct abundance of metabolic functions assigned to osmotic stress but there is insufficient evidence to suggest that the salinity, in these very dry islands, would be significantly different from the other locations. This apparent association of the bacterial community with osmotic stress might be simply due to the lack of other stresses in these relative pristine conditions. Dittami et al. (2016) showed that the capacity of *Ectocarpus* cultures to grow in diluted standard seawater medium is correlated with the presence of representatives of the Sphingomonadales order. This order is the best represented in all the replicates throughout all groups not allowing us to draw any direct link between these findings.

Overall, and besides the lack of replicates for metagenomics, our bacterial characterization through 16S barcoding is in agreement with metagenomic results. It is apparent that the environmental conditions (polluted, under anthropogenic influence coastal sites vs. offshore more pristine island waters) may shape not only the bacterial community composition, but consequently mirror the compositional differences in differentiated functional profiles. Our results suggested that the microbiome composition may primarily be influenced by the host traits (narrowing down from phylogenetic to individual host differences) and then by environmental conditions in which functional capability should be more important than bacterial taxonomic composition. A recent study (Hester et al., 2016) described that bacterial communities associated with seaweeds can be divided in two groups; the stable symbionts which are found specifically associated with a particular taxonomic group, and the sporadic symbionts which can be either the product of stochastic events or the response to environmental pressures (as supported by studies as Kelly et al., 2014). This suggests that changes in the symbiont members can lead to holobionts better adapted for particular conditions. These results are in line with our findings. Moreover, we hypothesize that community assembly through competitive lottery (as shown by Burke et al., 2011) may not be a simple gamble. From our results the bacterial communities found in both disturbed environments are not only known for the same set of functional capacities but are also phylogenetically related. So, this lottery may not only be restricted to the bacterial availability within the guild (where the ocean might be the source), but also to their ability to perform functions required under specific environmental demand. This might explain the larger number of OTUs (Figure S1) and metabolic similarities shared between the distinct *Asparagopsis* species from the same “disturbed” coastal environment when compared to the two conspecific, but in opposite environments, groups. Also, the Islands group showed reduced bacterial community richness when compared to the “disturbed environments” groups (Table S4). In agreement with other studies (Kelly et al., 2014; Marzinelli et al., 2015), we hypothesize that the host species might be the first factor shaping their bacterial community assembly. Yet, host-specific communities will likely adapt (as other traits) when facing environmental stresses by shifting to alternative communities (some specific bacteria and/or advantageous metabolic genes) that might act as *in situ* bioremediators to the host's advantage. As the environment changes, there is reassembling and the acquisition of new members.

However, in contrast to our descriptive study, experimental studies are required to evaluate the mechanisms behind these processes and assembly: Do hosts really select advantageous bacteria or is their abundance directly linked to their availability in the environment? Are those bacterial genes providing function to their macroalgal host by assuring defense against toxicity or are they just covering bacterial needs? The supportive metagenomic data in this study showed the importance of combining tools, 16S barcoding and metagenomics as well as metatranscriptomics in the future, to unveil the important factors shaping host-associated bacterial communities. Although, more detailed sampling with the inclusion of different replicates in the metagenomic data, as well as experimental and manipulative studies (concerning bacterial communities), are needed to determine the whole adaptive potential of seaweed species under climate change and anthropogenic influence. Holobionts should be understood as a dynamic unit (Hester et al., 2016), not a static group of genomes that evolve together as suggested by the Hologenome theory of evolution (Rosenberg et al., 2007).

## Author contributions

AE designed the research; AE, TA, and ES performed the sampling; TA performed the pyrosequencing and metagenomics analyses and analyzed the data; AE, TA, and ES co-wrote the paper.

## Funding

This work was supported by Fundação para a Ciência e a Tecnologia (FCT) through Net-Biome with the project SEAPROLIF (FCT Ref: NETBIOME/0001/2011), EXCL/AAG-GLO/0661/2012, and postdoctoral fellowships (SFRH/BPD/63703/2009 and SFRH/BPD/107878/2015) to AE.

### Conflict of interest statement

The authors declare that the research was conducted in the absence of any commercial or financial relationships that could be construed as a potential conflict of interest.

## References

[B1] Abbott I. A. (1999). Marine Red Algae of the Hawaiian Islands. Honolulu, HI: Bishop Museum Press.

[B2] Abraham W. R. Strömpl C. Meyer H. Lindholst S. Moore E. R. Christ R. (1999). Phylogeny and polyphasic taxonomy of Caulobacter species. Proposal of Maricaulis gen. nov. with Maricaulis maris (Poindexter) comb. nov. as the type species, and emended description of the genera Brevundimonas and Caulobacter. Int. J. Syst. Bacteriol. 49(Pt 3), 1053–1073. 10.1099/00207713-49-3-1053 10425763

[B3] Ainsworth T. Krause L. Bridge T. Torda G. Raina J.-B. Zakrzewski M.. (2015). The coral core microbiome identifies rare bacterial taxa as ubiquitous endosymbionts. ISME J. 9, 2261–2274. 10.1038/ismej.2015.39 25885563 PMC4579478

[B4] Aires T. Moalic Y. Serrao E. A. Arnaud-Haond S. (2015). Hologenome theory supported by co-occurrence networks of species-specific bacterial communities in siphonous algae (Caulerpa). FEMS Microbiol. Ecol. 91:fiv067 10.1093/femsec/fiv067 26099965

[B5] Aires T. Serrão E. A. Kendrick G. Duarte C. M. Arnaud-Haond S. (2013). Invasion is a community affair: Clandestine followers in the bacterial community associated to green algae, *Caulerpa racemosa*, track the invasion source. PLoS ONE 8:e68429. 10.1371/journal.pone.0068429 23874625 PMC3713043

[B6] Alain K. Tindall B. J. Intertaglia L. Catala P. Lebaron P. (2008). Hellea balneolensis gen. nov., sp. nov., a prosthecate alphaproteobacterium from the Mediterranean Sea. Int. J. Syst. Evol. Microbiol. 58, 2511–2519. 10.1099/ijs.0.65424-0 18984685

[B7] Alonso-Gutiérrez J. Figueras A. Albaigés J. Jiménez N. Viñas M. Solanas A. M.. (2009). Bacterial communities from shoreline environments (Costa da Morte, northwestern Spain) affected by the Prestige oil spill. Appl. Environ. Microbiol. 75, 3407–3418. 10.1128/AEM.01776-08 19376924 PMC2687268

[B8] Anast N. Smit J. (1988). Isolation and characterization of marine caulobacters and assessment of their potential for genetic experimentation. Appl. Environ. Microbiol. 54, 809–817. 16347590 10.1128/aem.54.3.809-817.1988 PMC202545

[B9] Andreakis N. Kooistra W. H. C. F. Procaccini G. (2009). High genetic diversity and connectivity in the polyploid invasive seaweed *Asparagopsis taxiformis* (Bonnemaisoniales) in the Mediterranean, explored with microsatellite alleles and multilocus genotypes. Mol. Ecol. 18, 212–226. 10.1111/j.1365-294X.2008.04022.x 19192176

[B10] Andreakis N. Procaccini G. Kooistra W. H. (2004). *Asparagopsis taxiformis* and *Asparagopsis armata* (Bonnemaisoniales, Rhodophyta): genetic and morphological identification of Mediterranean populations. Eur. J. Phycol. 39, 273–283. 10.1080/0967026042000236436

[B11] Andreakis N. Procaccini G. Maggs C. Kooistra W. H. C. F. (2007). Phylogeography of the invasive seaweed Asparagopsis (Bonnemaisoniales, Rhodophyta) reveals cryptic diversity. Mol. Ecol. 16, 2285–2299. 10.1111/j.1365-294X.2007.03306.x 17561891

[B12] Anonymous (2008). Plano de Ordenamento do Parque Natural Do Sudoeste Alentejano e Costa Vicentina - Fase 2 – Diagnóstico. Volume I/II. Lisbon: ICN-Instituto de Conservação da Natureza.

[B13] Barott K. L. Rodriguez-Brito B. Janouškovec J. Marhaver K. L. Smith J. E. Keeling P.. (2011). Microbial diversity associated with four functional groups of benthic reef algae and the reef-building coral *Montastraea annularis*. Environ. Microbiol. 13, 1192–1204. 10.1111/j.1462-2920.2010.02419.x 21272183

[B14] Biddanda B. Ogdahl M. Cotner J. (2001). Dominance of bacterial metabolism in oligotrophic relative to eutrophic waters. Limnol. Oceanogr. 46, 730–739. 10.4319/lo.2001.46.3.0730

[B15] Bray J. R. Curtis J. T. (1957). An Ordination of the upland forest community of southern Wisconsin.pdf. Ecol. Monogr. 27, 325–349. 10.2307/1942268

[B16] Bridle J. R. Vines T. H. (2007). Limits to evolution at range margins: when and why does adaptation fail? Trends Ecol. Evol. 22, 140–147. 10.1016/j.tree.2006.11.002 17113679

[B17] Burke C. Steinberg P. Rusch D. Kjelleberg S. Thomas T. (2011). Bacterial community assembly based on functional genes rather than species. Proc. Natl. Acad. Sci. U.S.A. 108, 14288–14293. 10.1073/pnas.1101591108 21825123 PMC3161577

[B18] Campbell A. H. Marzinelli E. M. Gelber J. Steinberg P. D. (2015). Spatial variability of microbial assemblages associated with a dominant habitat-forming seaweed. Front. Microbiol. 6:230. 10.3389/fmicb.2015.00230 25859245 PMC4374473

[B19] Caporaso J. G. Kuczynski J. Stombaugh J. Bittinger K. Bushman F. D. Costello E. K. (2010). Correspondence QIIME allows analysis of high- throughput community sequencing data Intensity normalization improves color calling in SOLiD sequencing. Nat. Publ. Gr. 7, 335–336. 10.1038/nmeth.f.303 PMC3156573 20383131

[B20] Chao A. (1984). Nonparametric estimation of the number of Classes in a population. Scand. J. Stat. 11, 265–270.

[B21] Chisholm J. R. M. Dauga C. Ageron E. Grimont P. A. D. Jaubert J. M. (1996). “Roots” in mixotrophic algae. Nature 381, 382–382. 10.1038/381382a0

[B22] Clarke K. R. Warwick R. M. (1994). Change in Marine Communities: An Approach to Statistical Analysis and Interpretation. Plymouth: Plymouth Marine Laboratory, Natural Environment Research Council.

[B23] Crump B. C. Koch E. W. (2008). Attached bacterial populations shared by four species of aquatic angiosperms. Appl. Environ. Microbiol. 74, 5948–5957. 10.1128/AEM.00952-08 18676705 PMC2565956

[B24] Dijoux L. Viard F. Payri C. (2014). The more we search, the more we find: discovery of a new lineage and a new species complex in the genus Asparagopsis. PLoS ONE 13:e103826 10.1371/journal.pone.0103826 25076489 PMC4116237

[B25] Dittami S. M. Duboscq-Bidot L. Perennou M. Gobet A. Corre E. Boyen C.. (2016). Host-microbe interactions as a driver of acclimation to salinity gradients in brown algal cultures. ISME J. 10, 51–63. 10.1038/ismej.2015.104 26114888 PMC4681850

[B26] Dittami S. M. Eveillard D. Tonon T. (2014). A metabolic approach to study algal-bacterial interactions in changing environments. Mol. Ecol. 23, 1656–1660. 10.1111/mec.12670 24447216

[B27] Dlugosch K. M. Parker I. M. (2008). Founding events in species invasions: genetic variation, adaptive evolution, and the role of multiple introductions. Mol. Ecol. 17, 431–449. 10.1111/j.1365-294X.2007.03538.x 17908213

[B28] Dring M. J. (2006). Stress resistance and disease resistance in seaweeds: the role of reactive oxygen metabolism. Adv. Bot. Res. 43, 176–207. 10.1016/S0065-2296(05)43004-9

[B29] European Environment Agency - EEA (2007). Halting the Loss of Biodiversity by 2010: Proposal for a First Set of Indicators to Monitor Progress in Europe. Technical Report 11/2007, European Environment Agency.

[B30] Egan S. Thomas T. Holmström C. Kjelleberg S. (2000). Phylogenetic relationship and antifouling activity of bacterial epiphytes from the marine alga *Ulva lactuca*. Environ. Microbiol. 2, 343–347. 10.1046/j.1462-2920.2000.00107.x 11200436

[B31] Haas B. J. Gevers D. Earl A. M. Feldgarden M. Ward D. V. Giannoukos G.. (2011). Chimeric 16S rRNA sequence formation and detection in Sanger and 454-pyrosequenced PCR amplicons. Genome Res. 21, 494–504. 10.1101/gr.112730.110 21212162 PMC3044863

[B32] Hanshew A. S. Mason C. J. Raffa K. F. Currie C. R. (2013). Minimization of chloroplast contamination in 16S rRNA gene pyrosequencing of insect herbivore bacterial communities. J. Microbiol. Methods 95, 149–155. 10.1016/j.mimet.2013.08.007 23968645 PMC4133986

[B33] Hester E. R. Barott K. L. Nulton J. Vermeij M. J. Rohwer F. L. (2016). Stable and sporadic symbiotic communities of coral and algal holobionts. ISME J. 10, 1157–1169. 10.1038/ismej.2015.190 26555246 PMC5029208

[B34] Hollants J. Leliaert F. Verbruggen H. Willems A. De Clerck O. (2013). Permanent residents or temporary lodgers: characterizing intracellular bacterial communities in the siphonous green alga Bryopsis. Proc. Biol. Sci. 280, 20122659. 10.1098/rspb.2012.2659 23303543 PMC3574326

[B35] Kelly L. W. Williams G. J. Barott K. L. Carlson C. A. Dinsdale E. A. Edwards R. A.. (2014). Local genomic adaptation of coral reef-associated microbiomes to gradients of natural variability and anthropogenic stressors. Proc. Natl. Acad. Sci. U.S.A. 111, 10227–10232. 10.1073/pnas.1403319111 24982156 PMC4104888

[B36] Khodjaniyazov K. U. (2012). Degradation and detoxification of persistent organic pollutants in soils by plant alkaloid anabasine. J. Environ. Prot. (Irvine,. Calif). 3, 97–106. 10.4236/jep.2012.31012

[B37] Lachnit T. Meske D. Wahl M. Harder T. Schmitz R. (2011). Epibacterial community patterns on marine macroalgae are host-specific but temporally variable. Environ. Microbiol. 13, 655–665. 10.1111/j.1462-2920.2010.02371.x 21078035

[B38] Lane D. J. (1991). 16S/23S rRNA sequencing, in Nucleic Acid Techniques in Bacterial Systematics, eds Stackebrandt E. Goodfellow M. (Chichester: John Wiley and Sons), 115–175.

[B39] Lee O. O. Chui P. Y. Wong Y. H. Pawlik J. R. Qian P.-Y. (2009). Evidence for vertical transmission of bacterial symbionts from adult to embryo in the Caribbean sponge Svenzea zeai. Appl. Environ. Microbiol. 75, 6147–6156. 10.1128/AEM.00023-09 19648378 PMC2753083

[B40] Mandrioli M. Manicardi G. C. (2013). Evolving aphids: One genome-one organism insects or holobionts? Invertebr. Surv. J. 10, 1–6. 27034285

[B41] Marcial Gomes N. C. Borges L. R. Paranhos R. Pinto F. N. Mendonça-Hagler L. C. S. Smalla K. (2008). Exploring the diversity of bacterial communities in sediments of urban mangrove forests. FEMS Microbiol. Ecol. 66, 96–109. 10.1111/j.1574-6941.2008.00519.x 18537833

[B42] Marzinelli E. M. Campbell A. H. Zozaya Valdes E. Vergés A. Nielsen S. Wernberg T. (2015). Continental-scale variation in seaweed host-associated bacterial communities is a function of host condition, not geography. Environ. Microbiol. 17, 4078–4088. 10.1111/1462-2920.12972 26148974

[B43] Massa S. I. Paulino C. M. Serrão E. A. Duarte C. M. Arnaud-Haond S. (2013). Entangled effects of allelic and clonal (genotypic) richness in the resistance and resilience of experimental populations of the seagrass *Zostera noltii* to diatom invasion. BMC Ecol. 13:39. 10.1186/1472-6785-13-39 24152760 PMC3818440

[B44] McKew B. A. Coulon F. Osborn A. M. Timmis K. N. McGenity T. J. (2007). Determining the identity and roles of oil-metabolizing marine bacteria from the Thames estuary, UK. Environ. Microbiol. 9, 165–176. 10.1111/j.1462-2920.2006.01125.x 17227421

[B45] Mellado M. Contreras R. A. González A. Dennett G. Moenne A. (2012). Copper-induced synthesis of ascorbate, glutathione and phytochelatins in the marine alga Ulva compressa (Chlorophyta). Plant Physiol. Biochem. 51, 102–108. 10.1016/j.plaphy.2011.10.007 22153245

[B46] Meyer F. Paarmann D. D'souza M. Olson R. Glass E. M. Kubal M. (2008). The metagenomics RAST server - a public resource for the automatic phylogenetic and functional analysis of metagenomes. BMC Bioinform. 9, 386 10.1186/1471-2105-9-386 PMC2563014 18803844

[B47] Morrow K. M. Ritson-Williams R. Ross C. Liles M. R. Paul V. J. (2012). Macroalgal extracts induce bacterial assemblage shifts and sublethal tissue stress in Caribbean corals. PLoS ONE 7:e44859. 10.1371/journal.pone.0044859 23028648 PMC3441602

[B48] Nakanishi K. Nishijima M. (1996). Bacteria that induce morphogenesis in *Ulva pertusa* (Chlorophyta) grown under axenic conditions. J. Phycol. 32, 479–482.

[B49] Neave M. J. Streten-Joyce C. Glasby C. J. McGuinness K. A. Parry D. L. Gibb K. S. (2012). The bacterial community associated with the marine Polychaete ophelina sp.1 (Annelida: Opheliidae) is altered by copper and zinc contamination in sediments. Microb. Ecol. 63, 639–650. 10.1007/s00248-011-9966-9 22038035

[B50] O'Hara A. M. Shanahan F. (2006). The gut flora as a forgotten organ. EMBO Rep. 7, 688–693. 10.1038/sj.embor.7400731 16819463 PMC1500832

[B51] Oliveira V. Gomes N. C. M. Cleary D. F. R. Almeida A. Silva A. M. S. Simões M. M. Q.. (2014). Halophyte plant colonization as a driver of the composition of bacterial communities in salt marshes chronically exposed to oil hydrocarbons. FEMS Microbiol. Ecol. 90, 647–662. 10.1111/1574-6941.12425 25204351

[B52] Oliveros J. C. (2007). VENNY. An Interactive Tool for Comparing Lists with Venn Diagrams. Available online at: http://bioinfogp.cnb.csic.es/tools/venny/index.html

[B53] Orole O. Adejumo T. (2011). Bacterial and fungal endophytes associated with grains and roots of maize. J. Ecol. Nat. Env. 3, 298–303.

[B54] Pauls S. U. Nowak C. Bálint M. Pfenninger M. (2013). The impact of global climate change on genetic diversity within populations and species. Mol. Ecol. 22, 925–946. 10.1111/mec.12152 23279006

[B55] Pinto E. Sigaud- Kutner T. C. S. Leitao M. A. S. Okamoto O. K. (2003). Review heavy metal – Induced oxidative stress in algae. J. Phycol. 39, 1008–1018. 10.1111/j.0022-3646.2003.02-193.x

[B56] Quast C. Pruesse E. Yilmaz P. Gerken J. Schweer T. Yarza P.. (2013). The SILVA ribosomal RNA gene database project: improved data processing and web-based tools. Nucleic Acids Res. 41, D590–D596. 10.1093/nar/gks1219 23193283 PMC3531112

[B57] Ratkevicius N. Correa J. A. Moenne A. (2003). Copper accumulation, synthesis of ascorbate and activation of ascorbate peroxidase in Enteromorpha compressa (L.) Grev. (Chlorophyta) from heavy metal-enriched environments in northern Chile. Plant Cell Environ. 26, 1599–1608. 10.1046/j.1365-3040.2003.01073.x

[B58] Rijstenbil J. W. Coelho S. M. Eijsackers M. (2000). A method for the assessment of light-induced oxidative stress in embryos of fucoid algae via confocal laserscan microscopy. Mar. Biol. 137, 763–774. 10.1007/s002270000443

[B59] Rosenberg E. Koren O. Reshef L. Efrony R. Zilber-Rosenberg I. (2007). The role of microorganisms in coral health, disease and evolution. Nat. Rev. Microbiol. 5, 355–362. 10.1038/nrmicro1635 17384666

[B60] Rosenberg E. Sharon G. Atad I. Zilber-Rosenberg I. (2010). The evolution of animals and plants via symbiosis with microorganisms. Environ. Microbiol. Rep. 2, 500–506. 10.1111/j.1758-2229.2010.00177.x 23766221

[B61] Sherwood A. R. (2008). Phylogeography of *Asparagopsis taxiformis* (Bonnemaisoniales, Rhodophyta) in the Hawaiian Islands: two Mtdna markers support three separate introductions. Phycologia 47, 79–88. 10.2216/07-39.1

[B62] Streftaris N. Zenetos A. (2006). Alien marine species in the Mediterranean - the 100 “worst invasives” and their impact. Mediterr. Mar. Sci. 7, 87–118. 10.12681/mms.180

[B63] Thompson J. R. Rivera H. E. Closek C. J. Medina M. (2015). Microbes in the coral holobiont: partners through evolution, development, and ecological interactions. Front. Cell. Infect. Microbiol. 4:176. 10.3389/fcimb.2014.00176 25621279 PMC4286716

[B64] Tonon T. Eveillard D. Prigent S. Bourdon J. Potin P. Boyen C.. (2011). Toward systems biology in brown algae to explore acclimation and adaptation to the shore environment. OMICS 15, 883–892. 10.1089/omi.2011.0089 22136637

[B65] Vincent P. Pignet P. Talmont F. Bozzi L. Fournet B. Guezennec J.. (1994). Production and characterization of an exopolysaccharide excreted by a deep-sea hydrothermal vent bacterium isolated from the polychaete annelid Alvinella pompejana. Appl. Environ. Microbiol. 60, 4134–4141. 16349441 10.1128/aem.60.11.4134-4141.1994 PMC201947

[B66] War A. R. Paulraj M. G. Ahmad T. Buhroo A. A. Hussain B. Ignacimuthu S.. (2012). Mechanisms of plant defense against insect herbivores. Plant Signal. Behav. 7, 1306–1320. 10.4161/psb.21663 22895106 PMC3493419

[B67] Zeng Y. Ma Y. Wei C. Jiao N. Tang K. Wu Z. (2010). Bacterial diversity in various coastal mariculture ponds in Southeast China and in diseased eels as revealed by culture and culture-independent molecular techniques. Aquac. Res. 41, e172–e186. 10.1111/j.1365-2109.2010.02499.x

[B68] Zilber-Rosenberg I. Rosenberg E. (2008). Role of microorganisms in the evolution of animals and plants: the hologenome theory of evolution. FEMS Microbiol. Rev. 32, 723–735. 10.1111/j.1574-6976.2008.00123.x 18549407

