# Supplementary material for: Host and Environmental Specificity in Bacterial Communities Associated to Two Highly Invasive Marine Species (Genus Asparagopsis)
Source: Front Microbiol. 2016 Apr 21;7:559. doi: 10.3389/fmicb.2016.00559 (PMC4839258; doi:10.3389/fmicb.2016.00559)
Supplement: Supplementary file 2 [file Table2.DOCX]

|  | ***A. armata* Mainland** | | | | | | | | | | | | | | | | ***A. taxiformis* Islands** | | | | | | | | ***A. taxiformis* Mainland** | | | | | | | |
| --- | --- | --- | --- | --- | --- | --- | --- | --- | --- | --- | --- | --- | --- | --- | --- | --- | --- | --- | --- | --- | --- | --- | --- | --- | --- | --- | --- | --- | --- | --- | --- | --- |
|  | *A. armata* Praia do Queimado | | | | | | *A. armata* Zambujeira do Mar | | | | | | *A. armata* Lagosteiros | | | | *A. taxiformis* Cape Verde | | | | *A. taxiformis* Madeira | | | | *A. taxiformis* Lagosteiros | | | | *A. taxiformis* Sines | | | |
| Vector # | 1PQA | 2PQA | 3PQA | 4PQA | 5PQA | 6PQA | 1ZMA | 2ZMA | 3ZMA | 4ZMA | 5ZMA | 6ZMA | Aa.1 | Aa.2 | Aa.3 | Aa.4 | AtCV1 | AtCV2 | AtCV3 | AtCV4 | AtM1 | AtM3 | AtM4 | AtM5 | At.1 | At.2 | LagAt1 | LagAt2 | At.Sin1 | At.Sin2 | SiAt1 | SiAt2 |
| **1** | 1 | 11 | 0 | 18 | 13 | 42 | 11 | 74 | 14 | 49 | 9 | 49 | 7 | 0 | 41 | 13 | 0 | 0 | 0 | 0 | 0 | 0 | 0 | 0 | 1 | 0 | 0 | 0 | 0 | 0 | 0 | 0 |
| **2** | 8 | 106 | 1 | 30 | 3 | 112 | 20 | 77 | 37 | 71 | 12 | 46 | 58 | 3 | 162 | 13 | 0 | 1 | 0 | 0 | 0 | 0 | 0 | 0 | 6 | 2 | 5 | 0 | 0 | 6 | 0 | 0 |
| **3** | 9 | 0 | 6 | 3 | 2 | 3 | 32 | 2 | 24 | 4 | 91 | 12 | 1 | 7 | 4 | 58 | 0 | 0 | 0 | 0 | 1 | 0 | 1 | 8 | 1 | 1 | 0 | 9 | 0 | 0 | 0 | 0 |
| **4** | 19 | 5 | 31 | 22 | 9 | 6 | 13 | 9 | 19 | 8 | 20 | 8 | 5 | 7 | 6 | 5 | 0 | 0 | 0 | 0 | 0 | 0 | 0 | 0 | 1 | 5 | 3 | 1 | 0 | 3 | 2 | 11 |
| **5** | 6 | 14 | 6 | 8 | 5 | 6 | 7 | 1 | 8 | 4 | 13 | 8 | 15 | 17 | 6 | 14 | 0 | 0 | 0 | 0 | 0 | 0 | 0 | 0 | 0 | 0 | 0 | 0 | 1 | 1 | 10 | 1 |
| **6** | 0 | 0 | 0 | 0 | 0 | 0 | 0 | 0 | 0 | 0 | 0 | 0 | 1 | 0 | 0 | 0 | 0 | 0 | 0 | 0 | 0 | 0 | 2 | 0 | 10 | 1 | 5 | 19 | 51 | 273 | 577 | 75 |
| **7** | 0 | 0 | 0 | 0 | 0 | 0 | 0 | 0 | 0 | 0 | 0 | 0 | 0 | 0 | 0 | 0 | 0 | 0 | 0 | 0 | 0 | 0 | 0 | 0 | 0 | 3 | 0 | 2 | 0 | 2 | 17 | 3 |
| **8** | 0 | 0 | 0 | 4 | 0 | 0 | 0 | 0 | 0 | 2 | 0 | 0 | 0 | 0 | 1 | 0 | 0 | 0 | 0 | 0 | 0 | 0 | 0 | 1 | 17 | 23 | 23 | 11 | 37 | 18 | 17 | 19 |
| **9** | 0 | 0 | 0 | 0 | 0 | 0 | 0 | 1 | 1 | 1 | 0 | 0 | 0 | 0 | 1 | 1 | 0 | 0 | 0 | 0 | 0 | 1 | 0 | 0 | 21 | 24 | 16 | 11 | 11 | 6 | 14 | 21 |
| **10** | 1 | 1 | 0 | 0 | 0 | 0 | 6 | 3 | 0 | 0 | 0 | 1 | 9 | 2 | 1 | 1 | 0 | 0 | 0 | 0 | 0 | 0 | 0 | 0 | 65 | 16 | 10 | 20 | 2 | 0 | 1 | 0 |
| **11** | 0 | 0 | 0 | 4 | 0 | 0 | 0 | 0 | 0 | 1 | 0 | 0 | 0 | 0 | 0 | 0 | 0 | 0 | 0 | 0 | 0 | 0 | 0 | 1 | 12 | 8 | 2 | 5 | 1 | 2 | 1 | 4 |
| **12** | 0 | 0 | 0 | 1 | 0 | 1 | 0 | 4 | 0 | 2 | 0 | 0 | 1 | 0 | 1 | 0 | 0 | 0 | 0 | 0 | 2 | 2 | 0 | 1 | 12 | 22 | 10 | 12 | 13 | 12 | 6 | 28 |
| **13** | 3 | 4 | 0 | 8 | 0 | 4 | 1 | 2 | 0 | 4 | 0 | 3 | 0 | 0 | 6 | 0 | 0 | 0 | 0 | 0 | 0 | 0 | 0 | 0 | 6 | 26 | 16 | 9 | 10 | 15 | 10 | 23 |
| **14** | 1 | 0 | 0 | 1 | 4 | 1 | 0 | 0 | 0 | 3 | 1 | 0 | 0 | 1 | 1 | 0 | 0 | 0 | 0 | 0 | 0 | 1 | 1 | 0 | 37 | 7 | 2 | 23 | 0 | 1 | 0 | 0 |
| **15** | 12 | 0 | 3 | 5 | 5 | 1 | 8 | 7 | 8 | 9 | 24 | 2 | 1 | 8 | 4 | 1 | 13 | 13 | 4 | 6 | 8 | 4 | 2 | 19 | 40 | 22 | 32 | 80 | 18 | 25 | 90 | 46 |
| **16** | 0 | 0 | 0 | 0 | 0 | 0 | 0 | 0 | 0 | 0 | 0 | 0 | 0 | 0 | 0 | 0 | 6 | 13 | 122 | 175 | 39 | 26 | 2 | 7 | 0 | 0 | 0 | 0 | 0 | 0 | 0 | 0 |
| **17** | 0 | 0 | 0 | 0 | 0 | 0 | 0 | 0 | 0 | 0 | 0 | 0 | 0 | 0 | 0 | 0 | 1 | 7 | 1 | 0 | 65 | 15 | 14 | 5 | 0 | 0 | 0 | 0 | 0 | 0 | 0 | 0 |
| **18** | 3 | 12 | 2 | 10 | 1 | 8 | 0 | 9 | 1 | 6 | 2 | 4 | 4 | 0 | 6 | 2 | 11 | 11 | 11 | 66 | 69 | 406 | 35 | 48 | 0 | 0 | 0 | 0 | 0 | 0 | 0 | 0 |
| **19** | 0 | 0 | 0 | 0 | 0 | 0 | 0 | 0 | 0 | 0 | 0 | 0 | 0 | 0 | 0 | 0 | 0 | 0 | 0 | 1 | 12 | 12 | 7 | 1 | 0 | 0 | 0 | 0 | 1 | 0 | 2 | 0 |
| **20** | 2 | 0 | 1 | 0 | 1 | 1 | 0 | 0 | 0 | 0 | 0 | 0 | 0 | 0 | 0 | 0 | 15 | 69 | 63 | 50 | 114 | 118 | 148 | 90 | 1 | 1 | 0 | 2 | 0 | 2 | 0 | 0 |
| **21** | 0 | 0 | 0 | 0 | 0 | 0 | 0 | 0 | 0 | 0 | 1 | 0 | 0 | 1 | 1 | 0 | 0 | 0 | 1 | 1 | 50 | 93 | 23 | 14 | 0 | 0 | 0 | 0 | 0 | 0 | 0 | 0 |
| **22** | 0 | 0 | 0 | 4 | 2 | 0 | 2 | 0 | 0 | 1 | 0 | 0 | 0 | 0 | 0 | 0 | 1 | 5 | 4 | 100 | 4 | 13 | 8 | 4 | 2 | 6 | 1 | 2 | 1 | 3 | 0 | 3 |

Table S2- **Abundance of OTUs, represented as vectors in Fig.3, for each replicate in each group** (these numbers were obtained from the OTU table after rarefaction and might not completely overlap the numbers in Table S1 – for easier consultation of these abundance values before rarefaction, the respective OTUs were highlighted in Table S1).
