# Supplementary figures and images for: Host and Environmental Specificity in Bacterial Communities Associated to Two Highly Invasive Marine Species (Genus Asparagopsis)
Source: Front Microbiol. 2016 Apr 21;7:559. doi: 10.3389/fmicb.2016.00559 (PMC4839258; doi:10.3389/fmicb.2016.00559)

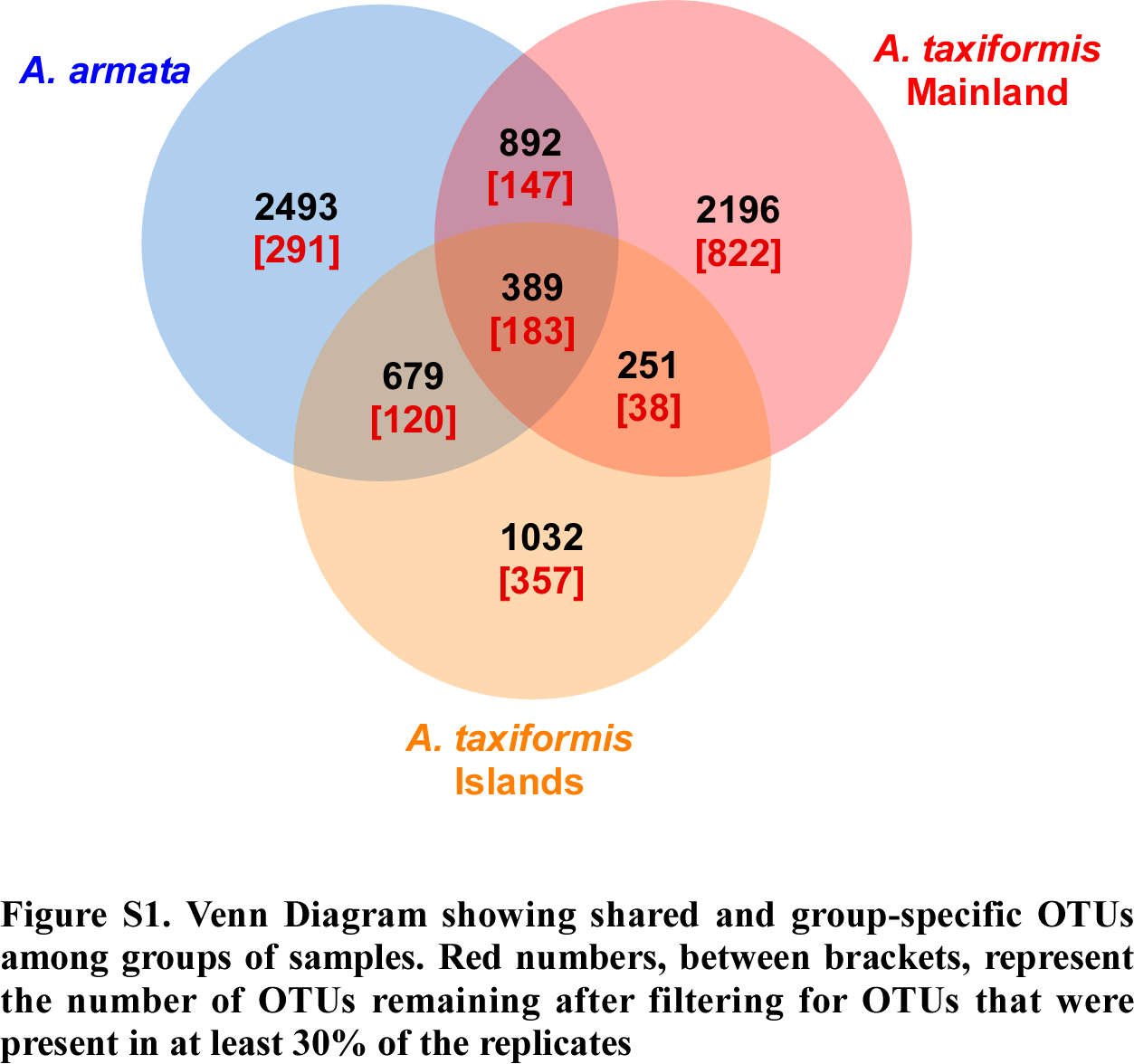

Supplement: Supplementary file 7 [file Image1.TIF]

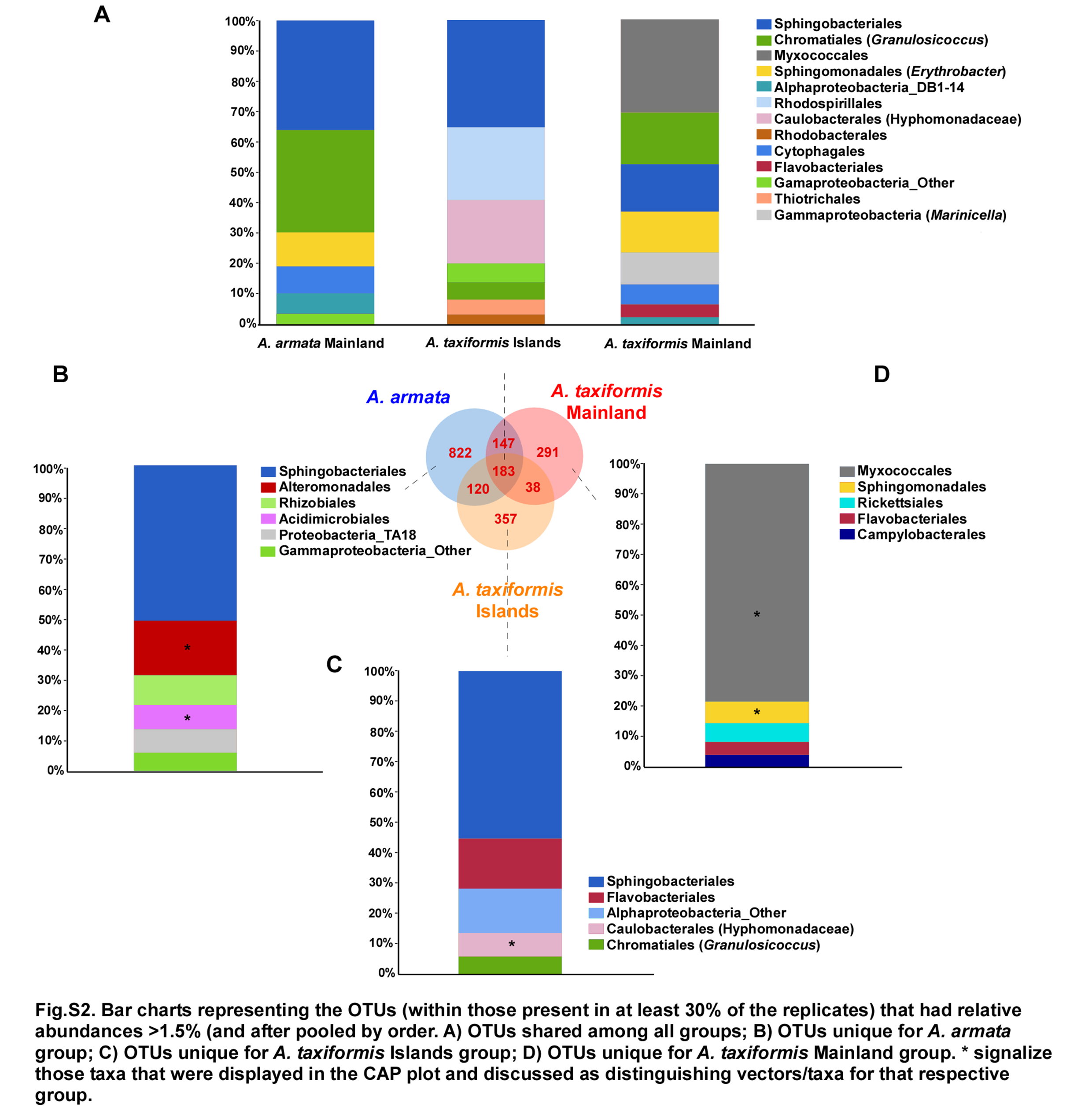

Supplement: Supplementary file 8 [file Image2.TIF]
